# Native *Trichoderma* species as multifunctional biocontrol agents for soil-borne disease management in groundnut and chickpea

**DOI:** 10.3389/fmicb.2026.1880339

**Published:** 2026-09-08

**Authors:** Dhruvi K. Suvagiya, Ramesh K. Kothari

**Affiliations:** Department of Biosciences, Saurashtra University, Rajkot, India

**Keywords:** biocontrol, multilocus sequencing, multitrophic interactions, phytobiome resilience, sustainable agriculture, *Trichoderma*

## Abstract

Sustainable agriculture increasingly relies on harnessing the complex, multitrophic network within the soil phytobiome to combat soil-borne pathogens. This study highlights the diversity and functional potential of *Trichoderma* spp. isolates from rhizospheric soils in the Saurashtra region, Gujarat of 250 soil samples, one hundred distinct *Trichoderma* isolates were obtained and further screened for their antagonistic efficacy against three major soil-borne fungal pathogens: *Sclerotium* spp., *Fusarium* spp. and *Macrophomina* spp. The *In vitro* assay found elite strains, particularly Tr79, Tr80 and Tr44, that consistently showed mycelial inhibition of up to 80%. Scanning electron microscopy (SEM) of Tr79 confirmed robust mycoparasitic interactions, including hyphal coiling and mycelial degradation. Molecular characterization using TEF1-*α* and RPB2 gene sequencing delineated these isolates into three clades: *T. harzianum*, *asperelloides* and *longibrachiatum*. Compatibility testing with 21 agrochemicals showed that isolates Tr79 and Tr80 were most resilient to most herbicides and insecticides, supporting their integration into integrated pest management. Field experiments with groundnut and chickpea showed that a split application of Tr79 enriched with Farmyard Manure (FYM) significantly improved germination and yield, boosting yield threefold compared with the control and reducing disease incidence to 10.15 and 17.63%, respectively. These findings highlight the potential of region-specific *Trichoderma* strains to enhance crop resilience and productivity through sustainable disease management. These findings are first regional Multilocus characterization from Saurashtra.

## 1 Introduction

Soil ecosystems are highly dynamic and functionally complex habitats in which microbes, plants and abiotic factors interact in deeply interconnected networks, influencing plant productivity and health. The phytobiome, composed of the plant and the microbial populations that surround it, are essential for controlling nutrient cycling, stress tolerance and disease prevention in these multitrophic systems (Chand Meena et al., 2018). A growing body of research indicates that exploiting beneficial plant-microbe interactions can provide a sustainable option for chemical-dependent agriculture, especially given soil-borne phytopathogens, which drastically impair agricultural output globally (Compant et al., 2019).

*Trichoderma* species have attracted global attention for their multifunctional role as biocontrol agents, plant growth boosters and systemic resistance inducers. These fungi exhibit several antagonistic behaviors, including mycoparasitism, competition for nutrients and space, the release of secondary metabolites and enzymes that degrade cell walls (Druzhinina et al., 2012). Furthermore, *Trichoderma’s* activation of plant defense pathways promotes physiological adaptability to biotic and abiotic stimuli, emphasizing their importance in sustainable crop management systems (Dubey and Patel, 2001).

*Trichoderma* spp.*’s* effectiveness as biocontrol agents is influenced by their genetic diversity, ecological adaptation and compatibility with agricultural techniques, especially in varied soil environments (Harman et al., 2004; Jaklitsch and Voglmayr, 2015). Extensive studies in India have shown that *Trichoderma* has a wide distribution and functional diversity across crops and agroecosystems; however, this diversity remains underexplored at the regional scale. Furthermore, the adaptation of native isolates to specific soil conditions, such as the alkaline soils widespread in portions of Gujarat, highlights the relevance of region-specific strain selection (Kumar Biswas and Sen, 2000). Despite this, agroecologically diverse places, such as Gujarat’s Saurashtra region, remain underexplored in terms of soil microbiome composition and *Trichoderma* variety.

Recent advances in molecular systematics have significantly improved the resolution of *Trichoderma* taxonomy. Multilocus sequencing approaches, particularly translation elongation factor 1-alpha (TEF1-*α*) and RNA polymerase II subunit (RPB2), provide robust frameworks for species delineation and phylogenetic placement (Kumari et al., n.d.). When combined with phenotypic and functional screening, these methods enable the exact selection of superior biocontrol candidates with more ecological relevance.

Despite the demonstrated efficacy of *Trichoderma* spp., their field performance is frequently unpredictable due to pesticide interactions, environmental variability and pathogen adaptation. Establish integrated disease management methods; it is critical to assess compatibility with routinely used agrochemicals and antagonistic potential *in vitro* and *in vivo* (Liu et al., *1999*). Furthermore, field validation under crop-specific conditions is essential for turning laboratory discoveries into actual agricultural applications.

In this context, the current work aims to isolate, characterize and evaluate indigenous *Trichoderma* spp. from Saurashtra’s rhizosphere soils. The study combines morphological, molecular, phylogenetic and functional investigations to select elite antagonistic isolates, assess their compatibility with agrochemicals and evaluate their field efficacy.

## 2 Materials and methodology

### 2.1 Sampling and isolation of *Trichoderma* strains

Total 250 rhizospheric soils were collected across eight districts of saurashtra region (Junagadh:21.52177°N,70.45683°E; Rajkot:22.30523°N,70.79245°E; Jamnagar:22.46900°N,70.05978°E; Amreli:21.60258°N,71.21795°E; Porbandar:21.64391°N,69.62891°E; Dwarka:22.24488°N,68.96887°E; GirSomanath:20.92304°N,70.66763°E; Surendranagar:22.72721°N,71.64004°E) Gujarat, India Samples were stored in sterilized zip-lock bags at 4 °C. One gram of soil was serially diluted to 10^−4^ and 100 μL aliquots were spread on potato dextrose agar (PDA) (Mitrović et al., 2023) for isolation. Morphologically distinct colonies were purified on PDA using the single-spore technique (Manzar et al., 2022) and kept on PDA slants at 4 °C.

#### 2.1.1 Pathogen source

The soil-borne phytopathogenic fungi *Sclerotium rolfsii*, *Fusarium oxysporum* and *Macrophomina phaseolina* used in this study were obtained as authenticated cultures from the culture collection maintained at the Main Oilseeds Research Station, Junagadh Agricultural University, Junagadh, Gujarat, India. The cultures were kindly donated by the station for research purposes. Prior to their use, the isolates had been previously isolated, purified, identified, and maintained under laboratory conditions. These pathogens were selected for the present investigation based on their confirmed pathogenicity and virulence against groundnut and chickpea under laboratory and field conditions.

### 2.2 Primary screening of *Trichoderma* isolates for antagonistic activity

*Trichoderma* isolates were evaluated against three phytopathogens *Fusarium oxysporum*, *Macrophomina phaseolina* and *Sclerotium rolfsii* by the dual plate culture technique. The mycelia disc of each pathogen measuring 5 mm was placed at the opposite ends of a PDA plate. The plates were incubated for 2 to 5 days at room temperature (28 ± 2 °C), after which, the antagonistic activity of *Trichoderma* spp. against phytopathogens was observed as zone of inhibition (ZOI) (Hajji-Hedfi et al., 2026a). The promising antagonistic activity against tested fungal pathogens were measured and the percent inhibition of average radial growth was calculated relative to the control as given equation and interactions were further examined using scanning electron microscopy (SEM).

I = (C-T/C) × 100.

Where, I = Inhibition per cent, C = Colony diameter in control plate, T = Colony diameter in treated plate (O’Gorman et al., 2009; Pedrero-Méndez et al., 2025).

### 2.3 Selection of elite isolates

Based on the inhibition percentages obtained against all three pathogens, five isolates (Tr79, Tr80, Tr44, Tr42 and Tr46) showing consistently superior antagonistic activity were selected for molecular characterization, agrochemical compatibility testing and field evaluation.

### 2.4 Molecular characterization of potent isolates

Genomic DNA of Five potent isolates were extracted from 7-day-old PDA cultures using the FastPrep®24 Tissue Homogenizer (MP Biomedicals GmbH, Germany). The translation elongation factor 1-alpha (TEF1-*α*) and RNA polymerase II second largest subunit (RPB2) gene regions were amplified using the primer pairs Tricho_728_F (5′-CATCGAGAAGTTCGAGAA GG-3′)/Tricho_1_LLE_rev (5′-AACTTGCAGGCAATGTGG-3′) (Nandini et al., 2021) and RPB2_5F (5′-GAYGAYMGWGATCAY TTYGG-3′)/RPB2_7CR (5′-CCCATRGCTTGYTTRCCCAT-3′) (Shoresh et al., 2010), respectively, under the recommended PCR conditions. Amplified products were purified using the FavorPrep™ PCR Purification Kit and sequenced bidirectionally using the BigDye® Terminator v3.1 Cycle Sequencing Kit on an Applied Biosystems SeqStudio Genetic Analyzer.

The obtained sequences were edited and compared with reference sequences in the NCBI database using BLASTn for species identification. Validated sequences were submitted to GenBank, and accession numbers were obtained for all isolates. Phylogenetic relationships were inferred using TEF1-*α* and RPB2 sequences together with reference *Trichoderma* sequences retrieved from GenBank. Multiple sequence alignment was performed using ClustalW, and phylogenetic trees were constructed in MEGA X using the Maximum Likelihood method with 1,000 bootstrap replicates.

### 2.5 Cultural compatibility with agrochemicals

Poisoned food technique was adopted for evaluation of Twenty-one different agrochemicals *in vitro*. Seven fungicides, viz. Carbendazim 25% + Flusilazole 12.5% SE, Carbendazim 12% + Mancozeb 63% WP, Prochloraz 5.7% + Tebuconazole 1.4% w/w ES, Metriram 55% + Pyraclostrobin 5%, Azostrobin 18.2% + Difenaconazole 11.4%, Carboxin 37.5% + Thiram 37.5% WS, Penflufen 13.28% w/w + Trifloxystrobin 13.28% w/w FS were screened at four different concentrations (0.01, 0.02, 0.03 and 0.04%), seven insecticides, viz. Chlorpyrifos 10%, Carbosulfan 25% EC, Cartap hydrochloric 50% SP, Fipronil 5% SC, Imidacloprid 600 FS, Carbofuran 3%, Pyripoxyfen 5% + Diufentron 25% EC were screened at four different concentrations (0.025, 0.05, 0.075 and 0.1%) and seven herbicides, viz. Quizalofop ethyl 5% EC, Sodium aceflurofen 16.5% + Clodinofloppropyl 8%, Propaquizafop 3.75% + Imazethapyr 2.5%, Fluzifop-p-butyl 13.4% + Fomesafen 11.1%, Pendinethalin 30% EC, Imazethpyr 35% + Imazamox 35%, Imazethapyr 10% SL were screened at four different concentrations (0.025, 0.05, 0.075 and 0.1%) against five potent isolates of *Trichoderma* to examine their inhibitory effect on the mycelial growth.

The requisite quantities of agrochemicals were incorporated into sterile molten PDA agar medium and shaken well to make it homogeneous. Medium (15 mL) was then poured into 90 mm sterile Petri dishes in three replicates for each treatment, which were allowed to solidify. Controls were maintained without any agrochemical. Each Petri dish was inoculated by placing 5 mm mycelial disc in center and incubated at 25 ± 1 °C for 2 days. The relative efficacies were known by the radial growth of the mycelium and examined their inhibitory effect after 2 days of incubation (Vandenkoornhuyse et al., 2015).

### 2.6 Field evaluation of selected *Trichoderma* isolates against stem rot and wilt under sick plot conditions

Field experiments were conducted during Kharif 2024 (groundnut) and Rabi 2024–25 (chickpea) at the Main Oilseeds Research Station, Junagadh Agricultural University, Junagadh, Gujarat, India (21.5° N, 70.5° E), located in the South Saurashtra Agro-climatic Zone VI. The experiment was laid out in a randomized block design (RBD) with three replications using the susceptible groundnut cultivar GG 20 and chickpea cultivar Gujarat Gram 5. Individual plots measured 5 × 5 m, with a spacing of 30 cm between rows and 10 cm between plants (Pedrero-Méndez et al., 2025; Woo et al., 2014).

The efficacy of five selected *Trichoderma* isolates was evaluated under sick plot conditions maintained for stem rot and wilt diseases. The experimental field was artificially infested with *Sclerotium rolfsii* and *Fusarium oxysporum f.* sp. *ciceri* by incorporating pathogen colonized sorghum grain inoculum into the soil at a rate of 50 g m^−2^ before sowing. The sick plot was maintained through periodic addition of pathogen inoculum to ensure uniform and adequate disease pressure throughout the experimental period.

The treatments consisted of two application schedules for each selected *Trichoderma* isolate. Treatments T1-T5 involved soil application of individual *Trichoderma* isolates at 2.5 kg ha^−1^ enriched in 250 kg FYM ha^−1^ and applied at sowing. Treatments T6-T10 involved soil application of the respective isolates at 1.25 kg ha^−1^ enriched in 125 kg FYM ha^−1^ applied at sowing and again at 30 days after sowing (DAS). Treatment T11 served as untreated control without Trichoderma application. Each treatment was replicated three times.

Observations on seed germination, stem rot incidence in groundnut, and wilt incidence in chickpea were recorded up to 90 days after sowing. Germination percentage was calculated as:

Germination (%) = (Number of seeds germinated/Total number of seeds sowing) × 100.

Disease incidence was calculated as:

Disease incidence (%) = (Number of infected plants/Total number of plants observed) × 100 (Hajji-Hedfi et al., 2026b).

At crop maturity, the produce from each plot was harvested, threshed, and weighed to determine yield. The recorded data were subjected to statistical analysis using analysis of variance (ANOVA), and treatment means were compared at the 5% level of significance.

### 2.7 Statistical analysis

All experiments were conducted with appropriate replication. Laboratory studies were analyzed using a completely randomized design (CRD), while field data were analyzed using a randomized block design (RBD). Data on percent mycelial inhibition, germination, and disease incidence were arcsine-transformed prior to analysis, while original means are presented for clarity. Treatment means were compared using Duncan’s Multiple Range Test (DMRT) at *p* ≤ 0.05. Agrochemical compatibility data were further analyzed using factorial ANOVA to assess interaction effects. The antagonism assay showed highly significant differences among treatments (*p* < 0.001), whereas field treatment assays revealed significant treatment effects (*p* < 0.005). Statistical analyses were performed using R software.

## 3 Results

### 3.1 Sampling, isolation, and identification of *Trichoderma* strains

**Figure 1:**
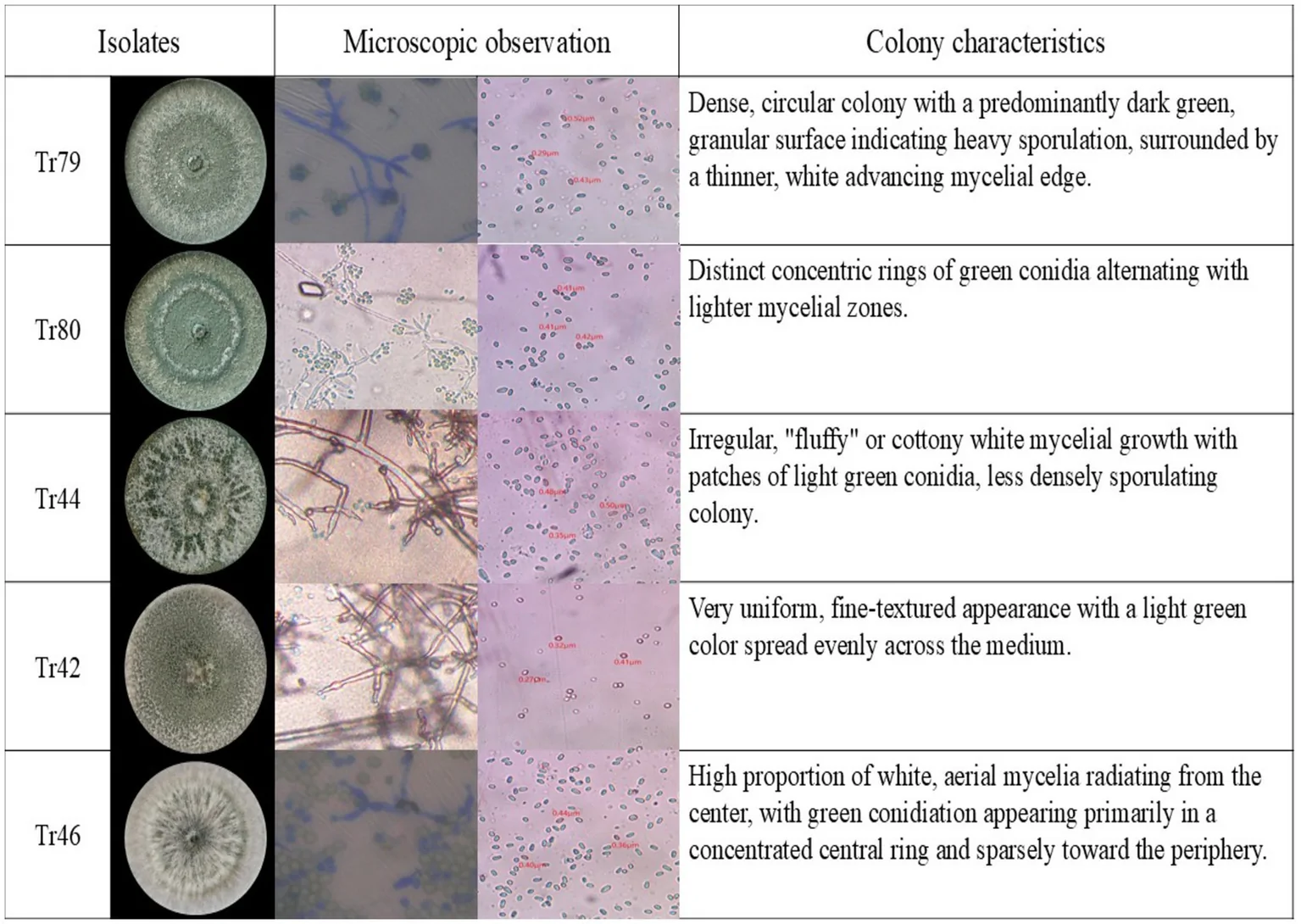
Colony morphology and microscopic observations of potent *Trichoderma* isolates.

Distinct one hundred fungal colonies were recovered from soil samples, and *Trichoderma* isolates were successfully identified through morphological and molecular characterization. Detail is mentioned in Figure 1.

#### 3.1.1 Molecular identification and sequence analysis

Molecular identification and partial sequencing of the translation elongation factor 1-alpha (TEF1-*α*) and RNA polymerase II second largest subunit (RPB2) genes were successfully performed for all five *Trichoderma* isolates (Tr79, Tr80, Tr44, Tr42, and Tr46) by using fungal universal primers RPB2_5F & RPB2_7CR (Jaklitsch and Voglmayr, 2015) for RPB2 amplification and Tr_728_F & Tr_1_LLE_rev (Liu et al., 1999) for TEF-*α* amplification. BLASTn analysis of the resulting sequences against the NCBI database revealed high similarities (94 to 99.75%) with reference *Trichoderma* spp., confirming genus-level identity and enabling preliminary species assignment. The generated sequences were deposited in GenBank under accession numbers PZ370521, PZ370522, PZ370524, PZ370526, PZ370529 for RPB2 and PZ370506, PZ370507, PZ370509, PZ370511, PZ370514 for TEF1-α.

Overall, the combined phylogenetic analysis of TEF1-α and RPB2 provided higher resolution than single-gene analysis, clearly delineating the isolates into three major species complexes: *harzianum*, *asperelloides*, and *longibrachiatum*. The high bootstrap values at major nodes further support the robustness of this multigene approach for accurate species-level identification within the genus *Trichoderma*.

### 3.2 *In vitro* dual culture assay

#### 3.2.1 Comparative *in vitro* antagonistic potential of *Trichoderma* isolates

**Figure 2:**
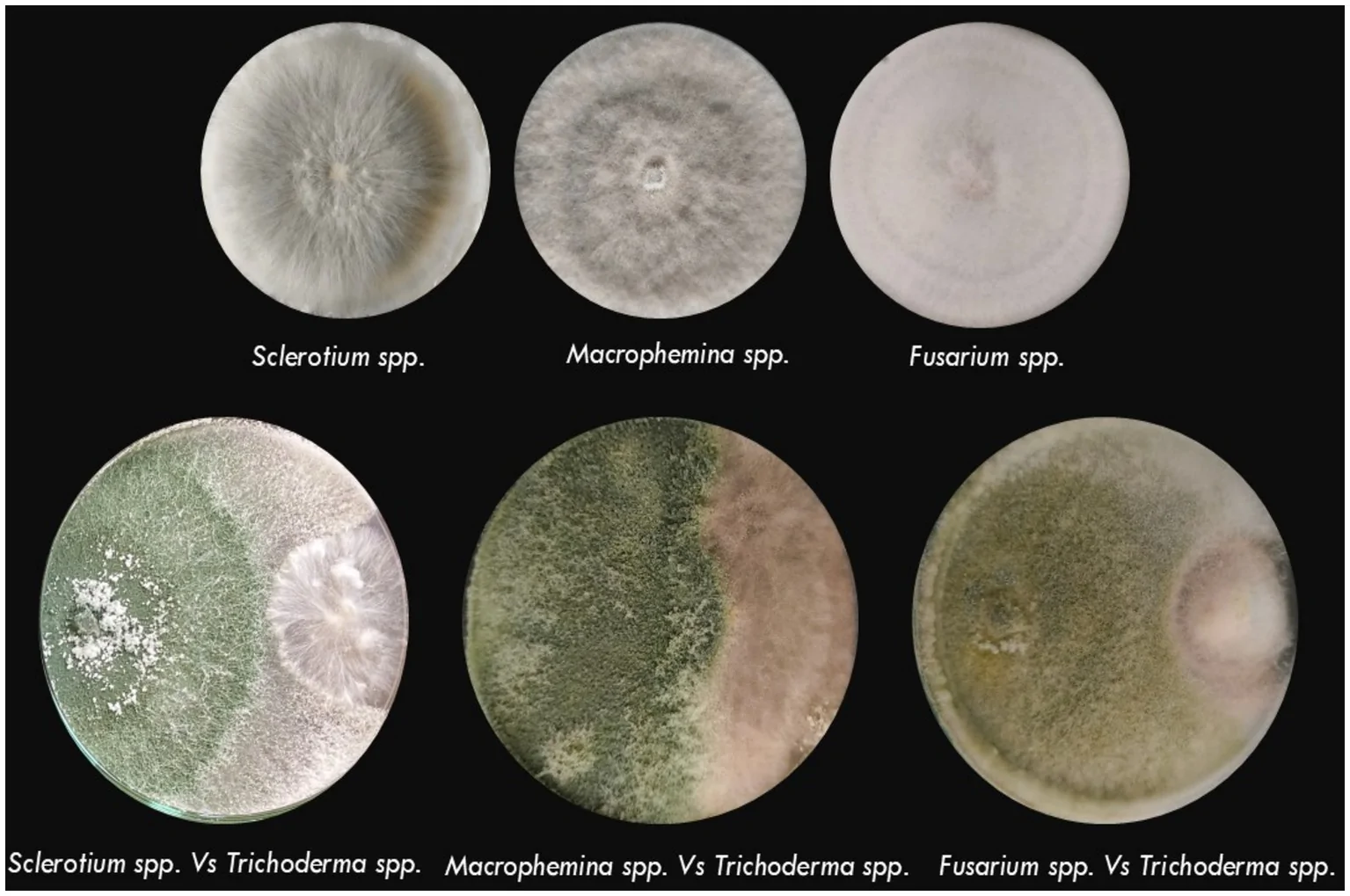
Antagonistic assay plates.

The dual-culture assay (Figure 2) revealed pronounced variability in antagonistic performance among the 100 *Trichoderma* isolates against *Macrophomina* spp. (MP), *Sclerotium* spp. (SC) and *Fusarium* spp. (FR). Two-way ANOVA confirmed that mycelial growth inhibition was significantly affected by both isolate identity (*F*₉₉,₁₉₈ = 1.65, *p* = 0.001) and pathogen species (*F*₂,₁₉₈ = 164.35, *p* < 0.001), indicating a strong interaction between isolate specific traits and pathogen susceptibility.

**Figure 3:**
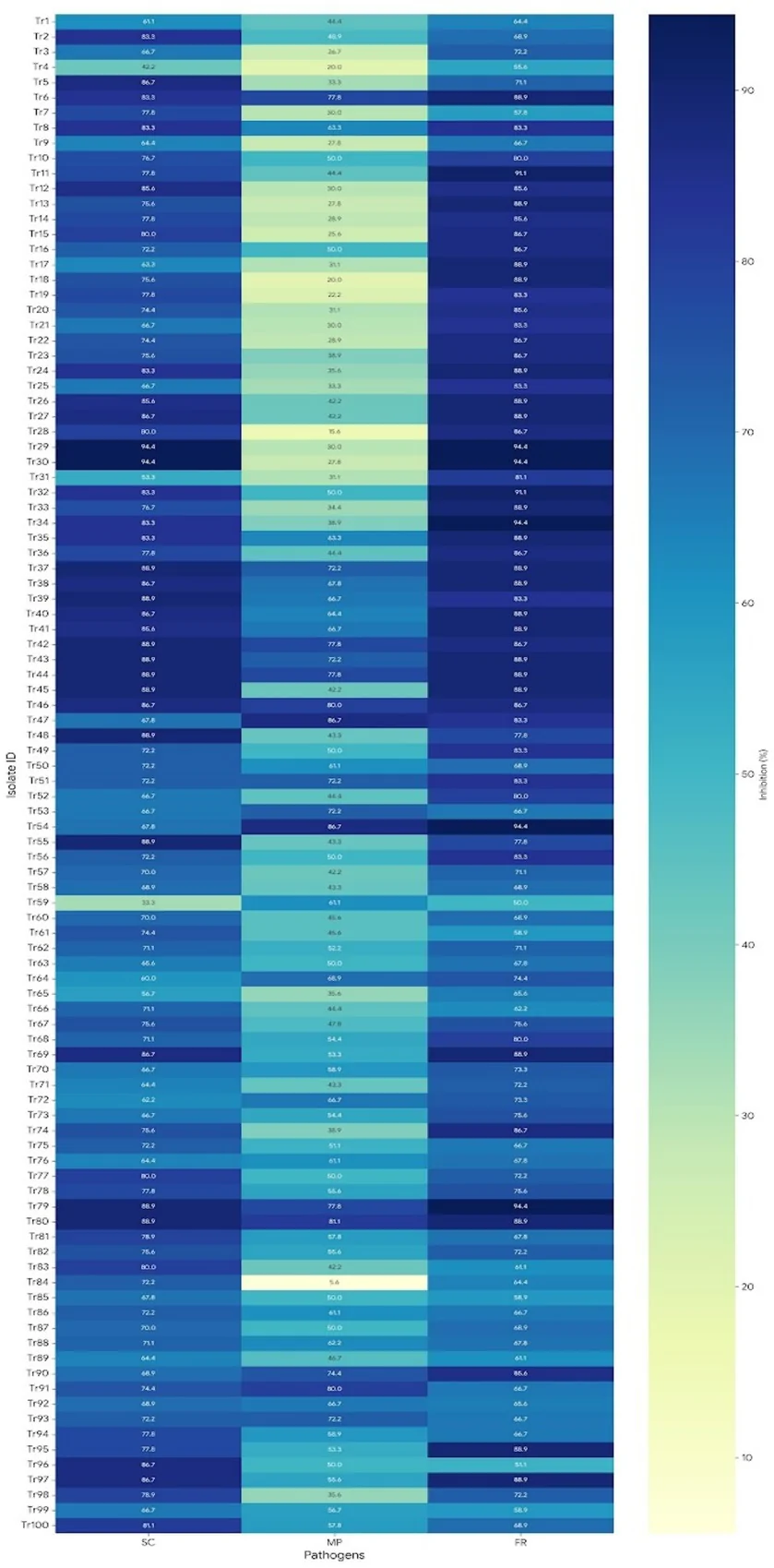
Heatmap of mycelial inhibition demonstrating the *in vitro* antagonistic efficacy of *Trichoderma* isolates against fungal pathogens.

Visual inspection of the inhibition heatmap further supported these statistical findings, revealing a consistent trend where inhibition against *Macrophomina* (MP) was comparatively lower, whereas *Sclerotium* (SC) and *Fusarium* (FR) exhibited higher inhibition levels (across most isolates (Figure 3). *Post hoc* Tukey’s HSD analysis corroborated this pattern, showing no significant difference between *Sclerotium* and *Fusarium* (*p* = 0.381), while *Macrophomina* remained significantly less susceptible (*p* < 0.001), with an approximate 25–30% reduction in inhibition.

#### 3.2.2 Selection of promising *Trichoderma* isolates based on broad-Spectrum antagonistic activity

Comparative analysis of pathogen inhibition data revealed substantial variability among *Trichoderma* isolates in their ability to suppress *Macrophomina phaseolina* (MP), *Sclerotium rolfsii* (SC), and *Fusarium oxysporum* f. sp. *ciceri* (FR). Isolates were evaluated based on their overall inhibition efficiency and consistency across all three pathogens. Among the tested isolates, Tr79, Tr80, Tr44, Tr42 and Tr46 consistently exhibited high levels of mycelial growth inhibition against each pathogen and clustered together in the heatmap region associated with strong antagonistic activity. Unlike many isolates that showed reduced effectiveness against *M. phaseolina*, these isolates maintained comparatively high inhibition across all three pathogens, demonstrating broad-spectrum antagonistic potential. Their consistent performance against multiple soil-borne pathogens indicates a high degree of adaptability and biocontrol efficacy, making them promising candidates for further evaluation under greenhouse and field conditions.

#### 3.2.3 Multivariate profiling: PCA and hierarchical clustering

**Figure 4:**
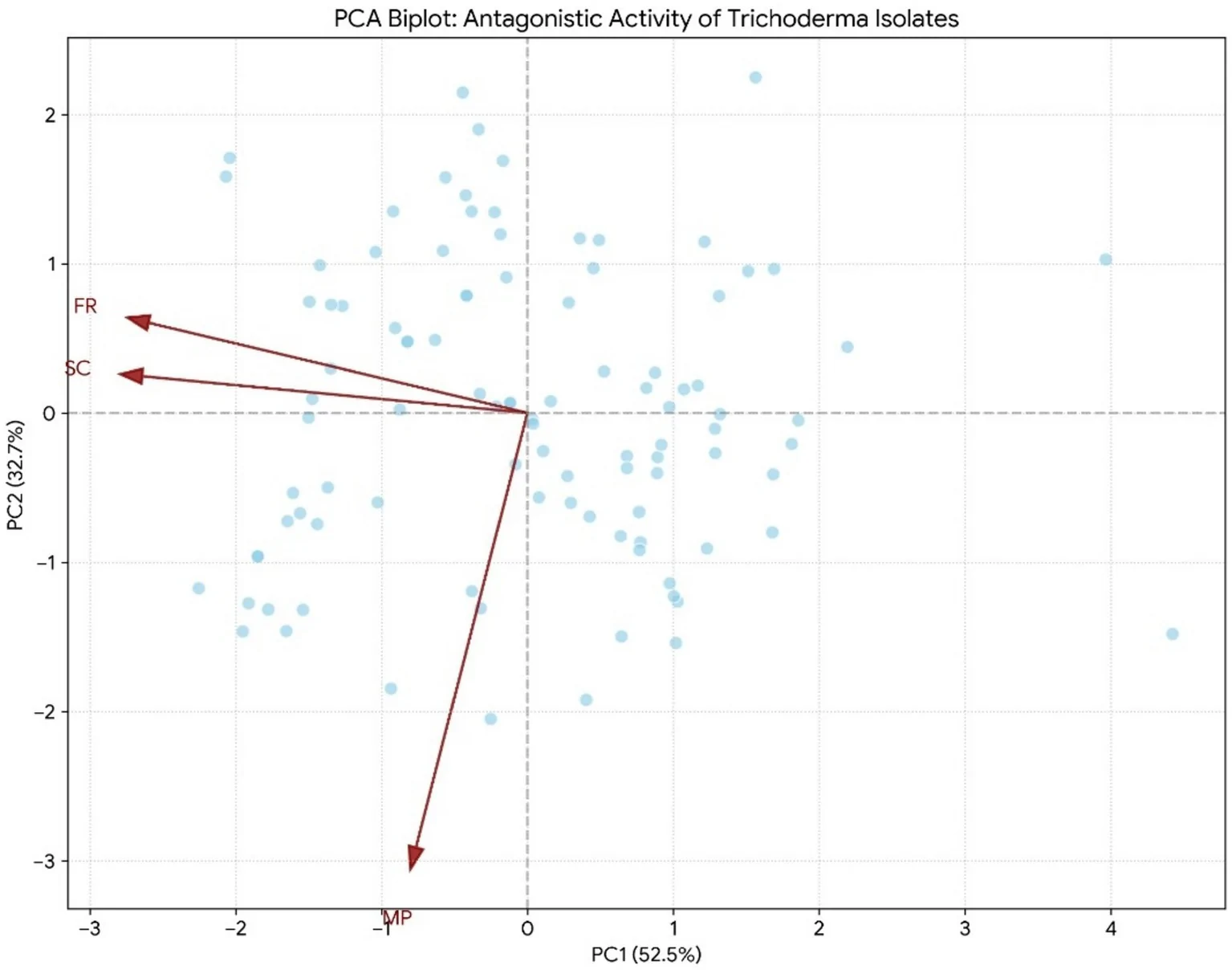
PCA biplot illustrates the clustering and correlation of *Trichoderma* isolates based on antagonistic activity against fungal pathogens.

Multivariate analyses, including PCA and hierarchical clustering, provided further insights into isolate behavior and pathogen-specific responses. The PCA biplot (Figure 4) showed that the responses of *Sclerotium* and *Fusarium* were closely aligned, indicating similar susceptibility patterns across isolates. In contrast, *Macrophomina* formed a distinct vector, reinforcing its differential response and relative resistance. Hierarchical clustering, visualized through the dendrogram-integrated heatmap, clearly segregated the isolates into three major functional groups:

- Group I (High-efficiency generalists): This cluster includes isolates such as Tr79, Tr80 and Tr44. These isolates demonstrated consistently high inhibition (>80%) and strong cross-pathogen efficacy.
- Group II (Moderate or differential performers): Isolates in this group showed high inhibition against SC and FR but reduced effectiveness against MP, indicating partial specificity or reduced activity against more resilient pathogens.
- Group III (Low-efficiency isolates): Characterized by low inhibition against MP and moderate activity against SC and FR, these isolates exhibited inconsistent antagonistic performance and limited practical applicability.

The clustering pattern highlights a clear gradient of antagonistic efficiency, with pathogen-dependent variability most evident in responses to *Macrophomina*.

#### 3.2.4 Scanning electron microscopy (SEM)

**Figure 5:**
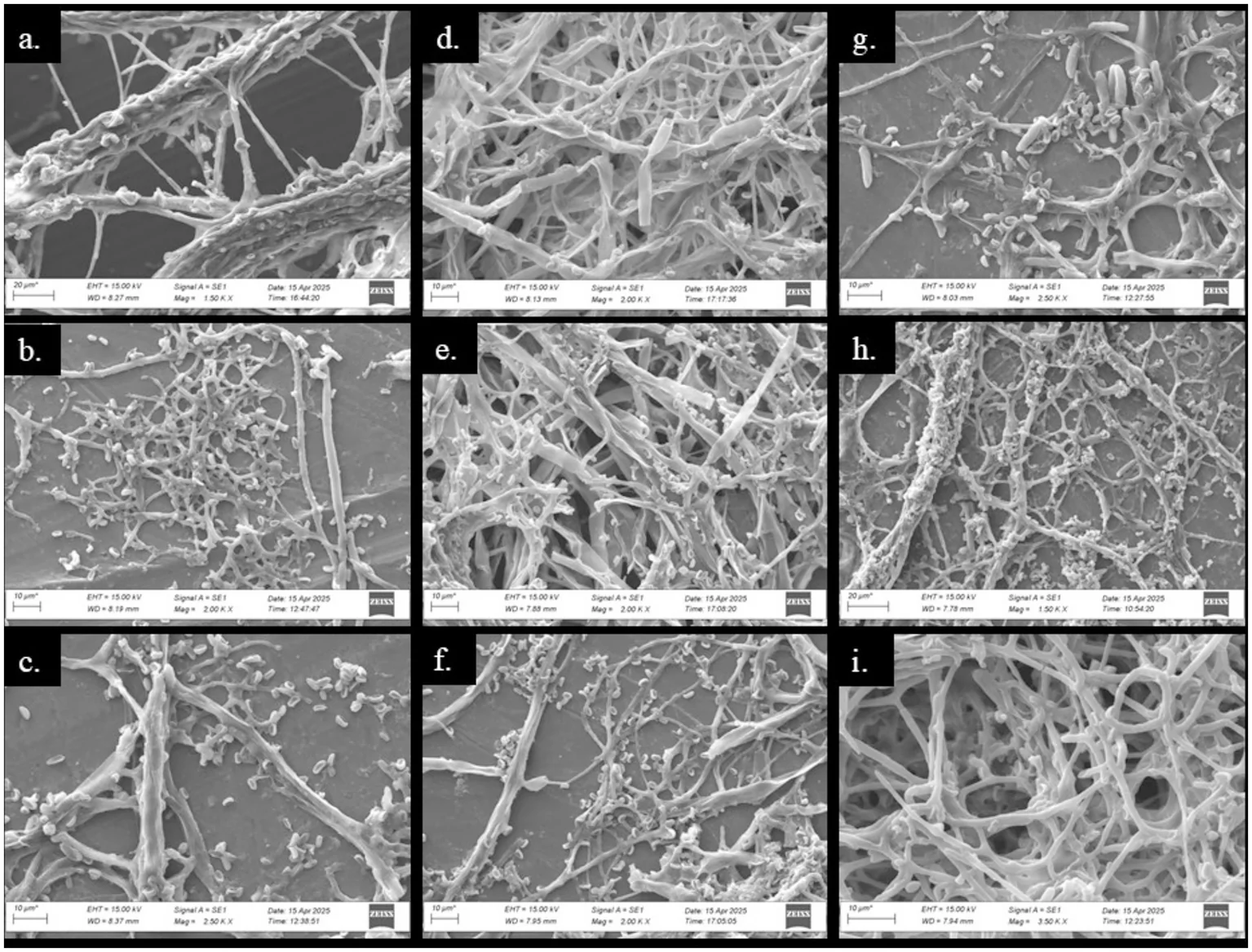
SEM images of Antagonistic Assay; (i) **a–c**- *Trichoderma vs. sclerotium* (ii) **d–f**-*Trichoderma vs Macrophomina* (iii) **g–i**- *Trichoderma vs. Fusarium.*

Scanning Electron Microscopy (SEM) analysis of the top-performing isolate Tr79 revealed strong mycoparasitic interactions, including extensive hyphal coiling, direct penetration and mycelial breakage. The pathogen hyphae exhibited severe ultrastructural damage, including cell wall pitting, deformation and cytoplasmic collapse. These features, as observed in SEM micrographs (Figure 5), confirm that the superior antagonistic activity of Tr79 is mediated by a combination of mycoparasitism.

### 3.3 Compatibility with agrochemicals

#### 3.3.1 *In vitro* sensitivity of *Trichoderma* isolates to multiclass agrochemicals

The compatibility of five *Trichoderma* isolates (Tr79, Tr80, Tr44, Tr42 and Tr46) was evaluated against a library of 21 agrochemicals, encompassing fungicides, insecticides and weedicides. One-way ANOVA revealed that mycelial inhibition was profoundly influenced by the chemical class and specific active ingredients (*F* = 104.13, *p* < 0.0001), while post-hoc analysis using Tukey’s HSD identified significant intra-isolate variations in tolerance levels.

#### 3.3.2 Differential toxicity across functional classes

A clear hierarchy of toxicity was observed across the tested categories. Broad-spectrum fungicides exhibited the highest suppressive pressure, with Carbendazim + Mancozeb and Prochloraz + Tebuconazole inducing complete mycelial arrest (100% inhibition) across all isolates and concentrations. These results categorize such fungicides as “Incompatible” for simultaneous field application with *Trichoderma*-based biopesticides. Notably, the combination of Penflufen + Trifloxystrobin stood out as a significant exception: isolates Tr79 and Tr80 showed remarkable resilience to this treatment, with mean inhibition rates of 12.3 and 7.3%, respectively, whereas Tr44 was extremely sensitive (81.9%).

In contrast, the weedicide and insecticide categories demonstrated significantly higher compatibility profiles. Most tested weedicides, particularly Imazethapyr 10% SL, proved non-toxic, with no growth inhibition across all isolates. Similarly, insecticides such as Carbofuran and Pyripoxyfen + Diufentron showed minimal impact on *Trichoderma* vitality (<10% inhibition), suggesting these can be seamlessly integrated into IPM (Integrated Pest Management) programs.

#### 3.3.3 Dose–response dynamics and superior isolate selection

Multivariate profiling and dose–response modelling identified Tr79 and Tr80 as superior “generalist” candidates for co-application. These isolates maintained high growth rates even at the maximum tested concentrations (400 ppm for fungicides and 1,000 ppm for others).

**Figure 6:**
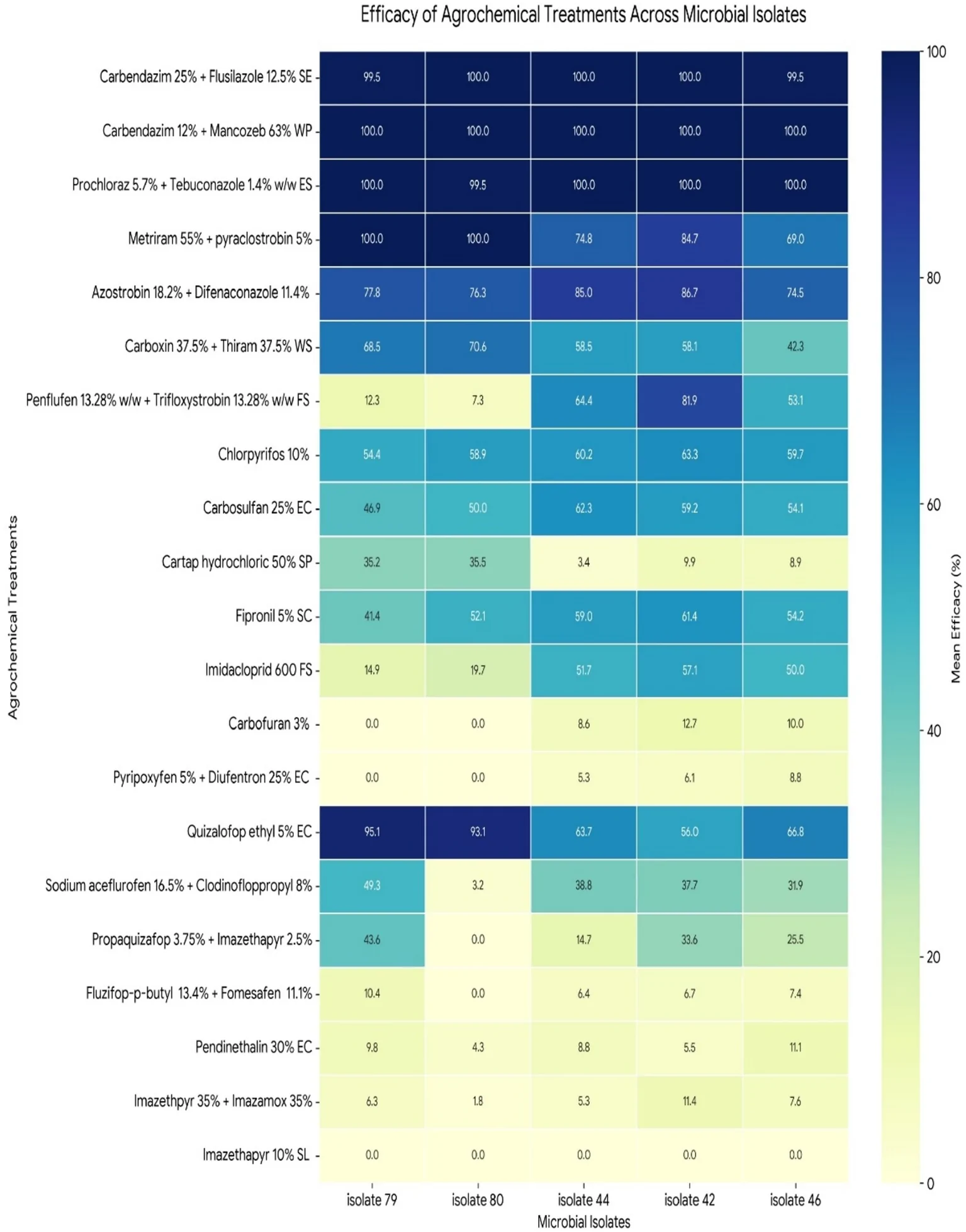
Heatmap of effect of agrochemicals on *Trichoderma* isolates in different concentration.

Hierarchical clustering and heatmap analysis categorized the interactions into three distinct compatibility zones (Figure 6). The “Highly Compatible” zone was dominated by weedicides and the isolates Tr79 and Tr80, which consistently outperformed Tr44 and Tr46 in terms of chemical tolerance. These findings suggest that Tr79 and Tr80 possess robust metabolic detoxification pathways or inherent cellular resilience, making them the most promising strains for commercial formulation in intensive agricultural systems where agrochemical use is prevalent.

### 3.4 Field evaluation

The field evaluation of groundnut and chickpea demonstrated that soil application of *Trichoderma* significantly improved plant stands, and productivity compared to the untreated control. Two-way ANOVA confirmed that both the isolate selection and the application timing played critical roles in field performance (*p* < 0.05) (see Table 1).

**Table 1:** Combine details of treatments and effect of germination (%), disease incidence (%) and yield (kg/ha) of groundnut and chickpea field experiments.

Sr. no.	Treatments	Germination (%)^*^		Disease incidence (%)^*^		Yield (kg/ha)^*^	
		Groundnut	Chickpea	Groundnut	Chickpea	Groundnut	Chickpea
1	SA of Tr79 @ 2.5 kg/ha enriched in 250 kg FYM /ha at the time of showing	84.00 (67.45)	74.20 (59.81)	10.17 (22.62)	35.53 (36.59)	2,459 (2,643)	1,813 (2,316)
2	SA of Tr80 @ 2.5 kg/ha enriched in 250 kg FYM /ha at the time of showing	82.33 (64.60)	64.63 (53.64)	12.27 (21.82)	39.11 (38.67)	2,221 (2,402)	1,840 (2,333)
3	SA of Tr44 @ 2.5 kg/ha enriched in 250 kg FYM /ha at the time of showing	80.67 (65.49)	57.96 (49.72)	11.79 (23.02)	46.43 (42.94)	2,087 (2,268)	1,667 (2,201)
4	SA of Tr42 @ 2.5 kg/ha enriched in 250 kg FYM /ha at the time of showing	73.33 (58.19)	56.17 (48.57)	13.35 (27.57)	47.95 (43.84)	1,823 (1,996)	1,650 (2,191)
5	SA of Tr46 @ 2.5 kg/ha enriched in 250 kg FYM /ha at the time of showing	71.87 (59.14)	55.85 (48.46)	11.53 (28.45)	48.53 (44.17)	1,481 (1,652)	1,556 (2,140)
6	SA of Tr79 @ 1.25 kg/ha enriched in 125 kg FYM /ha at the time of showing & 30 DAS	90.00 (72.53)	76.00 (61.15)	10.15 (18.92)	17.63 (24.76)	2,543 (2,735)	2,387 (2,660)
7	SA of Tr80 @ 1.25 kg/ha enriched in 125 kg FYM /ha at the time of showing & 30 DAS	83.33 (66.16)	75.46 (60.43)	12.48 (20.09)	18.61 (25.49)	2,438 (2,620)	2,070 (2,478)
8	SA of Tr44 @ 1.25 kg/ha enriched in 125 kg FYM /ha at the time of showing & 30 DAS	83.73 (66.47)	75.30 (60.72)	12.68 (20.67)	24.84 (29.59)	2,189 (2,373)	1,870 (2,343)
9	SA of Tr42 @ 1.25 kg/ha enriched in 125 kg FYM /ha at the time of showing & 30 DAS	78.53 (63.57)	64.39 (53.41)	13.37 (21.13)	30.66 (33.47)	1,948 (2,124)	1,757 (2,275)
10	SA of Tr46 @ 1.25 kg/ha enriched in 125 kg FYM /ha at the time of showing & 30 DAS	74.33 (60.08)	62.17 (52.19)	12.47 (24.79)	31.51 (34.15)	1,667 (1,838)	1,617 (2,177)
11	Control (untreated)	58.00 (49.67)	44.48 (41.81)	21.67 (44.19)	52.24 (46.31)	813 (987)	877 (1,556)
	S. Em.±	3.95	3.74	3.73	3.38	174.77	184.46
	CD at 5%	11.65	11.02	11.01	9.97	515.52	544.09
	CV%	9.87%	12.86%	18.62%	16.10	13.94%	15.21%

^*^FYM, Farmyard manure.

#### 3.4.1 Field efficacy of *Trichoderma* isolates on groundnut growth and disease suppression

**Figure 7:**
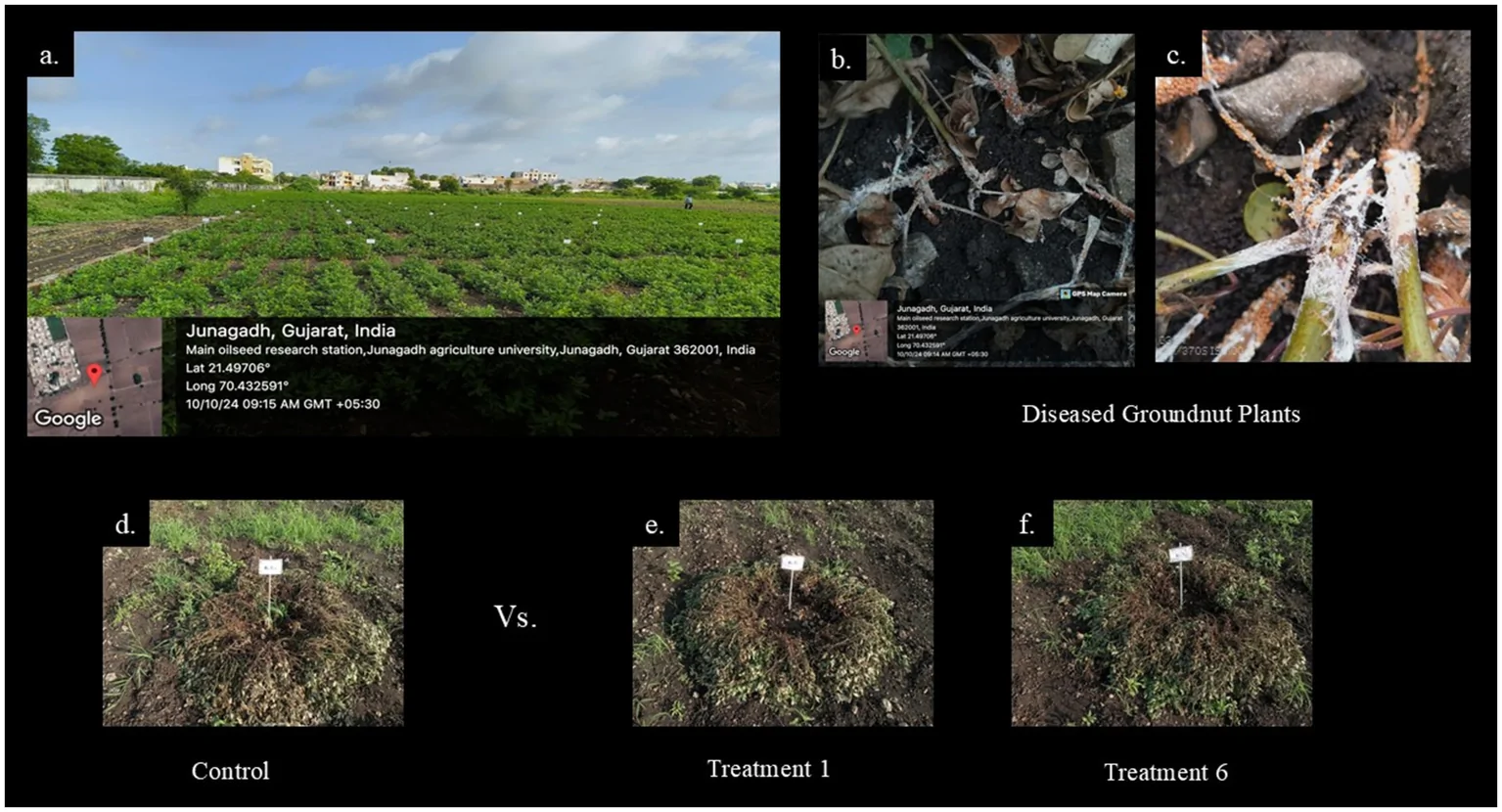
Groundnut Field Photographs. **(a)** Groundnut field layout, **(b** and **c)** Untreated/diseased control, **(d–f)** Control vs Treatments.

Among the treatments, the split application of Isolate Tr79 @ 1.25 kg/ha (enriched in FYM) at sowing and 30 Days After Sowing (DAS) yielded the most robust results. This treatment achieved a maximum germination rate of 90.00% and a peak pod yield of 2543.33 (kg/ha), representing a three-fold increase over the control (813.33 (kg/ha)). Furthermore, this regime was most effective in suppressing soil-borne pathogens, achieving the lowest disease incidence of 10.15%, which was statistically significantly lower than the control incidence of 21.67% (see Figure 7).

#### 3.4.2 Impact of *Trichoderma* enrichment on chickpea vitality and yield

In chickpea trials, the suppressive effect of *Trichoderma* against the wilt complex and other soil-borne diseases was highly pronounced. The integrated application of Isolate Tr79 @ 1.25 kg/ha (+30 DAS) again emerged as the superior treatment, providing a significant “booster effect” on crop health.

**Figure 8:**
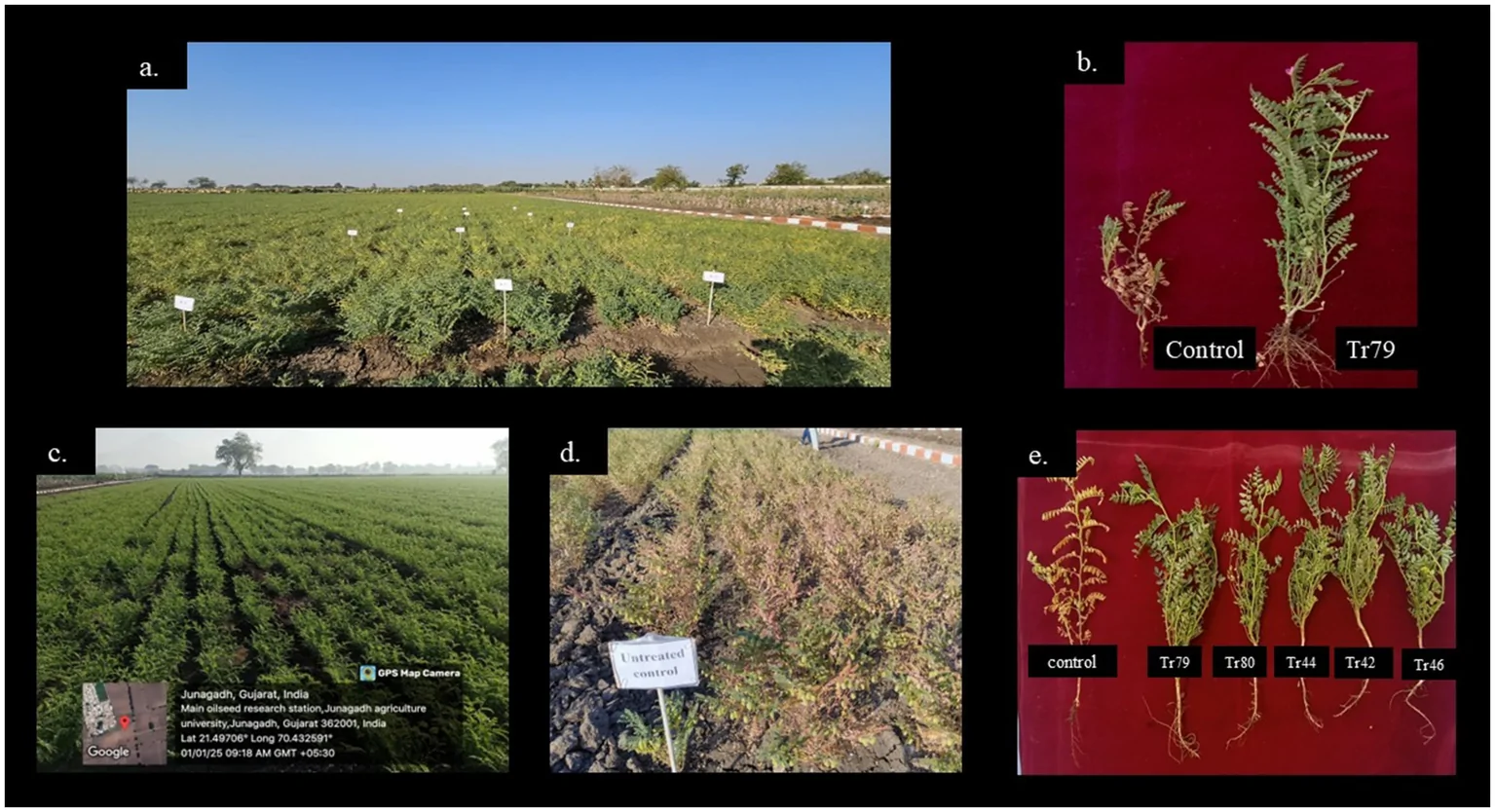
Chickpea Field Photographs. **(a, c)** Chickpea field layout, **(b, e)** Control vs Treatments, **(d)** Untreated/diseased control.

This treatment achieved a germination rate of 76.00%, significantly higher than the untreated control (44.48%). The most striking results were in disease reduction; while the control plots had a high disease incidence of 52.24%, plots treated with the split application of Isolate Tr79 reduced the incidence to just 17.63%. This superior disease management translated into a maximum grain yield of 2386.67 (kg/ha), outperforming the single high-dose basal application (2.5 kg/ha at sowing), which yielded 1813.33 (kg/ha) (see Figure 8).

#### 3.4.3 Comparative analysis of isolate performance and application frequency

Across both crops, a clear trend emerged in application frequency. The data suggest that enriching FYM with *Trichoderma* and providing a secondary application at 30 DAS is more effective than a single, higher-dose application at sowing.

Isolates Tr79 and Tr80 consistently showed higher field stability and rhizosphere competence than isolates Tr44, Tr42 and Tr46. The increased yield and decreased disease incidence in these treatments correlate with the superior antagonistic potential observed in previous *in vitro* assays, confirming that these isolates are highly effective candidates for large-scale field adoption in legume-based cropping systems.

## 4 Discussion

The present study demonstrates that indigenous *Trichoderma* isolates isolated from the rhizosphere soils of the Saurashtra region have a high potential for biological treatment of important soil-borne diseases affecting groundnut and chickpea. The superior performance of native isolates, notably Tr79, lends support to the idea that locally adapted *Trichoderma* populations have higher ecological fitness, rhizosphere competency and persistence in the field than non-native strains. Adapting to local environmental circumstances is crucial for successful biological management in various agroecosystems (Harman et al., 2004; Woo et al., 2014; Mitrović et al., 2023).

The isolate’s remarkable variance in antagonistic activity suggests significant functional diversity within indigenous *Trichoderma* populations. Tr79 consistently showed the greatest inhibition against *Fusarium oxysporum, Sclerotium rolfsii* and *Macrophomina phaseolina*, implying that effective antagonism is caused by the combined action of many mechanisms rather than a single mode of action. These methods involve rapid colonization of the rhizosphere, competition for nutrients and ecological niches, secretion of hydrolytic enzymes such chitinases, *β*-1,3-glucanases and proteases, formation of antifungal secondary metabolites, volatile chemical compounds and direct mycoparasitism. *Trichoderma* species exhibit multifunctional mechanisms (Benítez et al., 2004; Harman et al., 2004; Poveda, 2021). The relatively low inhibition of *M. phaseolina* observed in the current study is consistent with previous reports describing this pathogen as highly aggressive due to its abundant microsclerotia formation, broad host range and exceptional persistence under dry soil conditions, making complete biological suppression more difficult (Cayotopa-Torres et al., 2021; Hajji-Hedfi et al., 2026a, 2026b).

Scanning electron microscopy revealed direct evidence of mycoparasitic interactions, such as hyphal coiling, penetration and significant distortion of pathogen hyphae. These structural changes indicate enzymatic breakdown of fungal cell walls, confirming that direct parasitism plays a significant role in pathogen control. Previous research on *Trichoderma*-pathogen interactions has found that secreting cell wall-degrading enzymes disrupts pathogen integrity and inhibits fungal growth (Vinale et al., 2008; Mitrović et al., 2023). These findings lend credence to the idea that effective biological control is dependent on the combined action of enzymatic degradation, antimicrobial metabolites and competitive colonization.

Elite isolates were accurately identified using TEF1-*α* and RPB2 gene sequences, allowing for reliable species-level discrimination within *Trichoderma harzianum, T. asperelloides* and *T. longibrachiatum species* complexes. Morphological characteristics alone are frequently insufficient to distinguish closely related species, so multi-locus sequence analysis has become the acknowledged standard for *Trichoderma* taxonomy (Cai and Druzhinina, 2021).

Microbial biocontrol agents must be compatible with routinely used agrochemicals in order to be successfully integrated into integrated disease management (IDM) programmes. The varied reactions of *Trichoderma* isolates to fungicides, insecticides and herbicides found in the current investigation suggest that compatibility is mostly determined by the chemical nature and mechanism of action of each pesticide. Tr79 showed a higher tolerance to many agrochemicals, indicating that it might be used in conjunction with selective pesticides in the field. Hajji-Hedfi et al., 2026a, 2026b and other researchers found similar variability in fungicide tolerance among *Trichoderma* isolates, demonstrating that certain isolates retain viability and antagonistic activity even after exposure to compatible fungicides, facilitating their incorporation into IDM strategies.

Field trials indicated that Tr79 dramatically reduced disease incidence while increasing seed germination and crop yield in both groundnut and chickpea. Notably, the split treatment schedule outperformed a single application, demonstrating that having an active *Trichoderma* population during crop development improves rhizosphere colonization and provides long-term protection against soil-borne diseases. Aside from pathogen suppression, improved crop performance is most likely due to *Trichoderma’s* well-documented plant growth-promoting properties, which include increased nutrient solubilization, phytohormone production, systemic resistance induction and improved root architecture (Poveda, 2021; Woo et al., 2014). These findings are consistent with other recent field studies that demonstrate disease control and yield augmentation following the treatment of top *Trichoderma* strains under agricultural circumstances.

Although the current study effectively identified an efficient indigenous biocontrol isolate with high antagonistic activity, pesticide compatibility and field efficacy, the molecular basis of its antagonistic behavior was not experimentally examined. Future research that combines transcriptomics, comparative genomes, metabolomics and enzyme profiling will help us better understand the regulatory pathways that drive mycoparasitism, antimicrobial metabolite synthesis and plant-microbe interactions. Furthermore, multilocation field validation and large-scale formulation studies will be required to assess Tr79’s long-term stability and economic viability across various agroecological regions.

Overall, the current investigation shows that the indigenous isolate Tr79 has the ecological adaptability, broad-spectrum antagonistic activity, agrochemical compatibility and field performance needed for long-term treatment of key soil-borne diseases of groundnut and chickpea. The findings highlight the importance of using native rhizosphere microorganisms as ecologically friendly alternatives to chemical fungicides and they provide a solid scientific foundation for designing region-specific *Trichoderma*-based biocontrol formulations for sustainable agriculture.

## 5 Conclusion

This study highlights the potential of indigenous *Trichoderma* spp. as key components of sustainable crop protection and phytobiome management strategies. The integration of taxonomic characterization, functional screening, agrochemical compatibility assessment, and field validation created a comprehensive framework for developing effective biocontrol agents designed for local agroecosystems. The findings show that native Trichoderma populations can help to improve soil health, plant resilience, and minimize reliance on chemical disease management approaches, thereby promoting environmentally responsible agriculture. The study also underlines the significance of choosing locally adapted strains with multifunctional characteristics for successful field application and long-term ecological compatibility. These findings, taken together, increase the scientific basis for the development of Trichoderma-based bioinoculants customized to specific regions, as well as their incorporation into climate-resilient and sustainable disease management programs. Further study into formulation optimization, rhizosphere competency, metabolite profiling, and large-scale field validation will speed up the commercial deployment and widespread use of these beneficial microorganisms in modern agriculture.

## Data Availability

The datasets presented in this study can be found in online repositories. The names of the repository/repositories and accession number(s) can be found in the article/Supplementary material.
